# Mutations in *RECQL* Gene Are Associated with Predisposition to Breast Cancer

**DOI:** 10.1371/journal.pgen.1005228

**Published:** 2015-05-06

**Authors:** Jie Sun, Yuxia Wang, Yisui Xia, Ye Xu, Tao Ouyang, Jinfeng Li, Tianfeng Wang, Zhaoqing Fan, Tie Fan, Benyao Lin, Huiqiang Lou, Yuntao Xie

**Affiliations:** 1 Key Laboratory of Carcinogenesis and Translational Research (Ministry of Education), Breast Center, Peking University Cancer Hospital & Institute, Beijing, the People’s Republic of China; 2 State Key Laboratory of Agro-Biotechnology, College of Biological Sciences, China Agricultural University, Beijing, the People’s Republic of China; Women's College Research Institute, CANADA

## Abstract

The genetic cause for approximately 80% of familial breast cancer patients is unknown. Here, by sequencing the entire exomes of nine early-onset familial breast cancer patients without *BRCA1/2* mutations (diagnosed with breast cancer at or before the age of 35) we found that two index cases carried a potentially deleterious mutation in the *RECQL* gene (RecQ helicase-like; chr12p12). Recent studies suggested that *RECQL* is involved in DNA double-strand break repair and it plays an important role in the maintenance of genomic stability. Therefore, we further screened the *RECQL* gene in an additional 439 unrelated familial breast cancer patients. In total, we found three nonsense mutations leading to a truncated protein of RECQL (p.L128X, p.W172X, and p.Q266X), one mutation affecting mRNA splicing (c.395-2A>G), and five missense mutations disrupting the helicase activity of RECQL (p.A195S, p.R215Q, p.R455C, p.M458K, and p.T562I), as evaluated through an *in vitro* helicase assay. Taken together, 9 out of 448 *BRCA*-negative familial breast cancer patients carried a pathogenic mutation of the *RECQL* gene compared with one of the 1,588 controls (*P* = 9.14×10^-6^). Our findings suggest that *RECQL* is a potential breast cancer susceptibility gene and that mutations in this gene contribute to familial breast cancer development.

## Author Summary

In this study, we aimed to find novel breast cancer susceptibility genes by whole-exome sequencing in nine early-onset familial breast cancer patients without *BRCA1/2* mutations. We found that two index cases carried a potentially deleterious mutation in the *RECQL* gene (RecQ helicase-like). We further screened the *RECQL* gene in an additional 439 unrelated familial breast cancer patients. In total, we found nine index cases carried a pathogenic mutation in the *RECQL* gene among the 448 *BRCA*-negative familial breast cancer patients. The *RECQL* is one of five RecQ helicase proteins (named RECQL, BLM, WRN, RECQL4 and RECQL5). *RECQL* is considered to be genome caretaker, and mutations in three of five RecQ genes, *BLM*, *WRN* and *RECQ4* are associated with cancer predisposition and/or premature aging. Here, we are the first to report that mutations in the *RECQL* gene are associated with predisposition to breast cancer and this finding may have potential clinical implications and raise research questions about *RECQL*.

## Introduction

Breast cancer is the most common malignancy disease in women world wide. Among these, approximately 10% of breast cancer patients have a family history of breast cancer (referred as familial breast cancer), but only 10–15% of familial breast cancer is owing to germline mutations in one of the two high penetrance breast cancer susceptibility genes—*BRCA1* and *BRCA2*[1]. Additionally, mutations in the moderate breast cancer susceptibility genes, such as *PALB2*, *ATM*, *CHEK2*, *BRIP1* and *RAD51C*, contribute to 5% of familial breast cancers[2–6]. Therefore, the genetic causes of approximately 70–80% familial breast cancer remain to be discovered.

Recently, next-generation sequencing assay provides a new platform to find cancer susceptibility genes. To find potential breast cancer susceptibility genes, we performed whole-exome sequencing in nine early-onset familial breast cancer patients who do not carry a germline mutation in the *BRCA1/2* genes; all nine cases were diagnosed with breast cancer at or before the age of 35, the index case had at least one first-degree relative affected with breast cancer. Based on the data of whole-exome sequencing of the nine cases, we further screened the potential gene in an additional 439 unrelated familial breast cancer patients without *BRCA1/2* mutations and performed *in vitro* functional analyses to evaluate whether the mutations disrupt the function of the potential gene. Finally, our results indicate that *RECQL* (RecQ helicase-like) is a novel breast cancer susceptibility gene.

## Results

The detail information of the nine unrelated early-onset familial breast cancer patients who were subjected to whole-exome sequencing is presented in S1 Table. Approximately 46,000 variants were identified by whole-exome sequencing in each sample. We filtered the data for genes that harbored novel, heterozygous rare variants that were truncating mutations or splice-site variants. We further retained only genes that contain different variants shared in two or more cases (S2 Table).

As a result, there were three genes that fit these criteria and were validated by Sanger sequencing: *TTLL2*, *VSIG2* and *RECQL* (S3 Table). Of these genes, *RECQL* is of great interest because it is involved in DNA repair process. The two mutations in the *RECQL* gene were found in two index cases through the whole-exome assay (S1A and S1B Fig), one index case carried a nonsense mutation in exon 4 of the *RECQL* gene (encoding L128X), leading to a premature stop codon; another index case carried a non-synonymous mutation (R539P) that was predicted to affect the function of *RECQL*.

To determine the germline mutations in the *RECQL* gene in an additional cohort of familial breast cancer patients, we screened the entire coding region of *RECQL* gene using Sanger sequencing assays in 439 familial breast cancer patients without *BRCA1/2* mutations. In total, we found 15 germline variants in the *RECQL* gene in the 448 familial breast cancer patients (including the nine index cases for whole-exome sequencing) (Table 1 and S1 Fig). These variants contained three nonsense mutations (L128X, W172X and Q266X), two potential splice-site mutations (c.395-2 A>G and c.868-12_868-11del) and ten missense mutations (M1T, S63P, A195S, R215Q, N363S, R455C, M458K, R539P, H461R, and T562I) (Table 1). We further tested the germline mutations in the *RECQL* gene in 1,588 healthy controls, and three missense mutations (M1T, N363S and H461R) were found in the controls (Table 1). The three missense mutations (M1T, N363S and H461R) were present in both familial breast cancer patients and controls. Among these mutations, the mutation frequency of M1T (rs146924988) and N363S (rs138663409) was similar between familial breast cancer patients and controls. Thus, these two variants are more likely to be neutral (Table 1).

**Table 1:** Mutations in *RECQL* gene identified in the 448 *BRCA1/2*-negative familial breast cancer patients.

Coding mutation^a^	Protein change	Predictive algorithms		Helicase activity	LOH in tumors^d^	Non-*BRCA* FBC n = 448	Controls n = 1588
		SIFT^b^	PolyPhen^c^				
383T>G	L128X	—	—	ND	ND	1	0
516G>A	W172X	—	—	ND	ND	1	0
796C>T	Q266X	—	—	ND	(-)	1	0
395-2A>G	G132fs*	—	—	ND	(-)	1	0
644G>A	R215Q	No	Pb	Completely lost	ND	1	0
1363C>T	R455C	Yes	Pb	Completely lost	ND	1	1
1373T>A	M458K	Yes	Ps	Completely lost	(-)	1	0
1685C>T	T562I	Yes	Pb	Completely lost	(-)	1	0
583G>T	A195S	No	No	Partially lost	(-)	1	0
1616G>C	R539P	Yes	Ps	Normal	ND	1	0
187T>C	S63P	No	No	Normal	ND	1	0
1382A>G	H461R	Yes	Pb	Normal	ND	1	1
2T>C	M1T	Yes	No	ND	ND	4	9^e^
1088A>G	N363S	No	No	ND	ND	3	7
868–12_868-11del	—	—	—	ND	ND	1	0

FBC, familial breast cancer; ND, not detected.

^a^The reference transcript is *RECQL* transcript 001 (ENST00000444129);

^b^SIFT: Yes, affects function; No, tolerated.

^c^PolyPhen: Pb, probably damaging; Ps, possibly damaging; No, benign;

^d^(-), a retained WT allele;

^e^n = 748.

**Fig 1:**
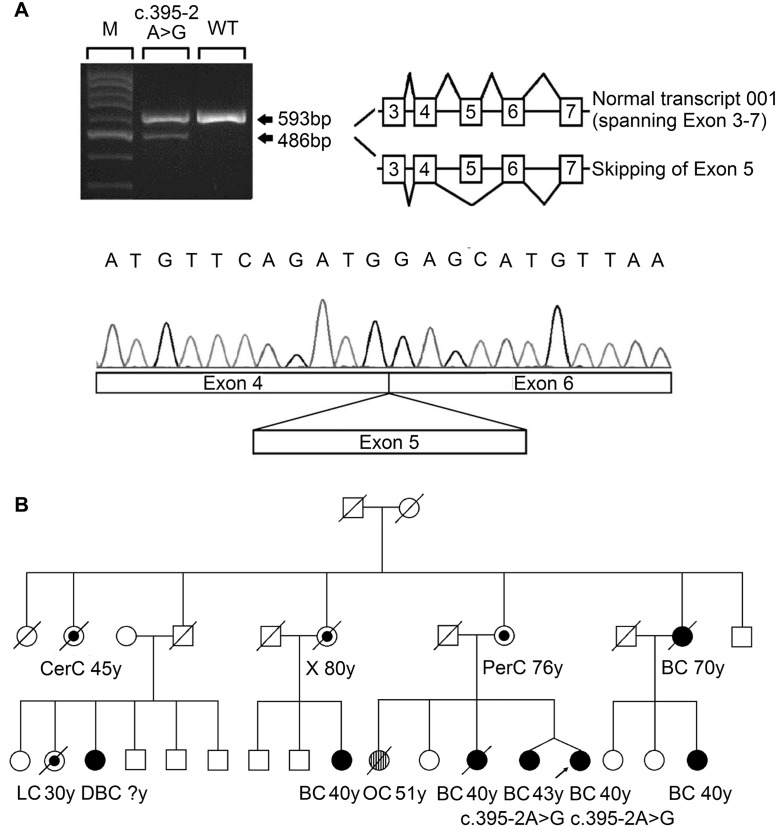
Analysis of the splicing mutation 395–2 A>G. (A) RT-PCR analysis of RNA extracted from the blood sample of the affected index case, using primers located in exon 3 and exon 7. The PCR products showed a reduced normal transcript (ENST00000444129) and an additional aberrant splicing transcript skipping exon 5, as confirmed by Sanger sequencing. (B) The pedigree of the index case who carried the *RECQL* c.395-2 A>G mutation. Types of cancer and age in years (y) at first diagnosis are given underneath each symbol. Filled circles represent breast cancer (BC) and double breast cancer (DBC); streaked circles represent ovarian cancer (OC); centre circles represent other types of cancer (CerC, cervical cancer; LC, lung cancer; PerC, peritoneal cancer; X, uninformative cancer).

The three nonsense mutations (L128X, W172X and Q266X) lead to premature protein termination, and therefore, we considered these to be clearly pathogenic. The two splice-site mutations were predicted to affect the splice site. RNA from peripheral blood samples was isolated from the index case who carried the 395-2A>G mutation. The RT-PCR pattern of the *RECQL* gene from the 395-2A>G mutation carrier revealed a reduced expression of the normal transcript and one new truncated product compared to the control. Further sequencing confirmed that the 395–2 A>G mutation resulted in exon 5 skipping in the abnormal RT-PCR product (Fig 1A). This product of the abnormal transcript disrupts the helicase domain of RECQL and leads to a premature stop signal (G132fs*); therefore, the 395-2A>G mutation is pathogenic. The pedigree of the index case who carried the *RECQL* 395-2A>G mutation is presented in Fig 1B. Both the index and the twin, who were negative for *BRCA1/2* mutations, carried the mutation; they had the disease at the ages of 40 and 43, respectively. Additionally, at least ten individuals in this family had breast/ovarian cancer, lung cancer, cervical cancer, or peritoneal cancer (Fig 1B). Unfortunately, blood samples were only available for the twin; thus, it was not possible to perform the segregation analysis in this family. Another index carried the c.868-12_868-11del mutation; however, RT-PCR analysis revealed that this mutation did not affect the splicing of *RECQL* (S2 Fig). Thus, the c.868-12_868-11del mutation was neutral.

To investigate whether the remaining eight missense mutations (S63P, A195S, R215Q, R455C, M458K, H461R, R539P, and T562I) influenced the efficacy of RECQL helicase activity, we first examined the structures of the RECQL protein (RCSB PDB, 2V1X and 2WWY) (S3 Fig). On the basis of the crystal structure, T562 is located in a β-hairpin, which is required for DNA unwinding [7,8]; A195 is involved in dimer interaction[8]; R215 is located near the ADP-binding pocket and is expected to weaken ATP hydrolysis[9]; the conserved residues R455 and M458 are located in the zinc binding subdomain, which is important for whole-protein stability[10](S3 Fig).

**Fig 2:**
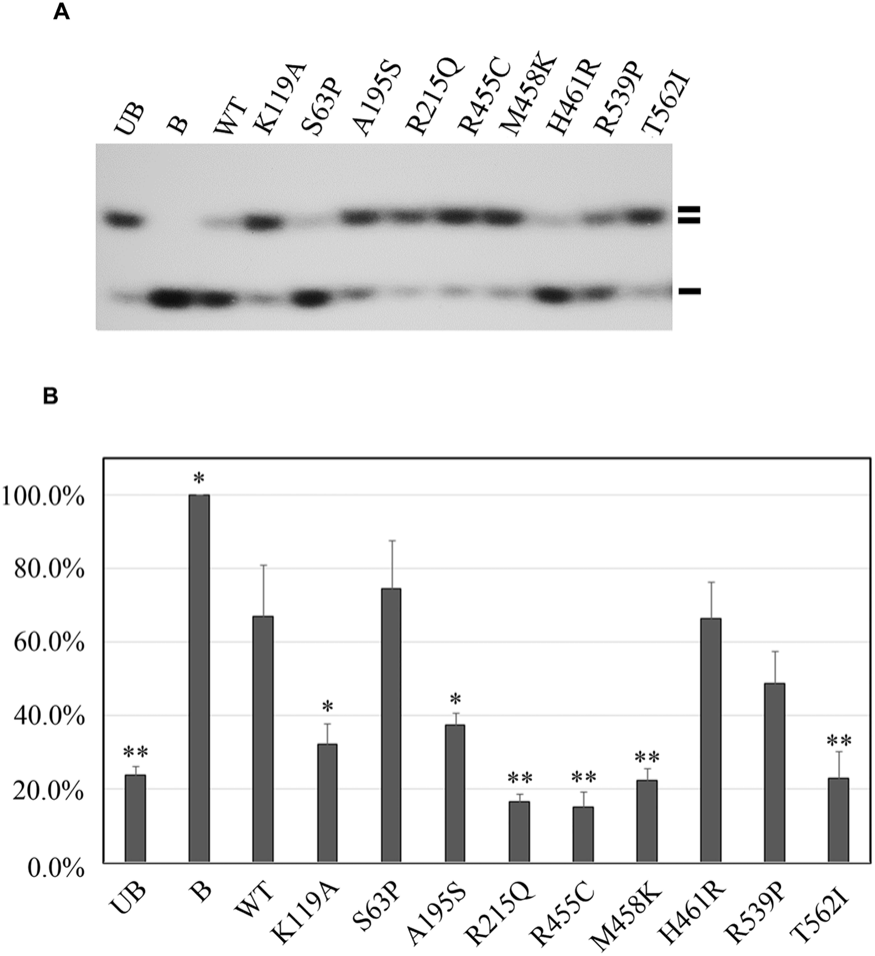
Functional analyses of the eight missense mutations in the *RECQL* gene. (A) helicase activity of the eight *RECQL* missense mutants. The helicase-defective K119A mutant was included as a negative control. B, boiled dsDNA; UB, unboiled dsDNA; WT, wild type; double lines, dsDNA; single lines, ssDNA. (B) Quantitative analysis of the helicase assay of RECQL mutants. Helicase data represent the mean of three independent experiments with the mean±S.D. indicated by error bars. The percent unwinding of mutants were compared to the WT level. The results of an unpaired, two-tailed t-test for the mutants are shown. *p < 0.05; **p <0.01.

Next, we performed helicase activity assays using GST fusion proteins *in vitro*[11]. The K119A mutation served as a negative control that is reported to affect the helicase activity of RECQL[12]. By assessing the helicase ability to unwind forked DNA substrates, single-strand DNA was essentially undetectable with four mutations, R215Q, R455C, M458K, and T562I, suggesting that these four mutations completely disrupted the helicase activity (Fig 2A and 2B). Additionally, we found that the A195S mutant lost approximately 83.4% of the helicase activity compared to the wild-type of RECQL. The remaining three missense mutations (S63P, H461R and R539P) showed similarly effective helicase activities compared to the wild-type of RECQL (Fig 2A and 2B). Thus, the mutations of S63P, H461R and R539P were neutral. Taken together, five missense mutations (R215Q, R455C, M458K, T562I and A195S) were pathogenic.

**Fig 3:**
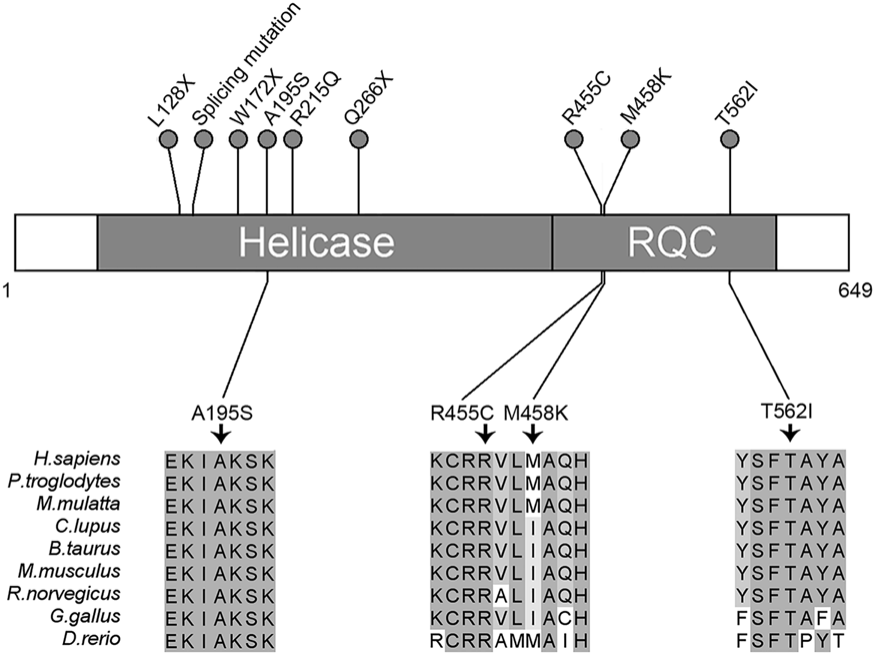
Schematic representation of *RECQL* pathogenic mutations. Two domains of RECQL are shown: The helicase domain (amino acids 63–418) and the RQC domain (amino acids 419–592). The nonsense, missense and splicing mutations are designated with a circle at the representative protein position. The splicing mutation is referred to as *RECQL* c.395-2A>G. The bottom part of the figure shows the four highly conserved mutations. Higher percentage identity at a given amino acid position is indicated.

In total, nine germline mutations in the *RECQL* gene in the nine familial breast cancer patients were identified as pathogenic, including three nonsense mutations (L128X, W172X and Q266X), one splice-site mutation (395-2A>G) and five missense mutations (A195S, R215Q, R455C, M458K and T562) (Table 1 and Fig 3). The pedigrees of the nine families are present in Fig 1B and S4 Fig. The average number of the breast cancer cases in the nine families were 2.8 cases/per family, with a mean age of onset of 47.8 years. The overall frequency of the *RECQ*L germline mutations in the 448 familial breast cancer patients without *BRCA1/2*mutations was 2.0% (9 of 448). One pathogenic mutation R455C was found in the 1,588 controls. The prevalence of the *RECQL* germline mutations was significantly higher in familial breast cancer patients than in the controls (9/448 vs. 1/1,588; the Fisher exact test, *P* = 9.14×10^–6^).

We then analyzed the clinical information of the nine cases with *RECQL* pathogenic mutations (S4 Table). The mean age at diagnosis of breast cancer in the nine cases with *RECQL* mutation was younger than in those without *RECQL* mutation (45.1vs.51.3 years; *P* = 0.12). The hormone receptor and HER2 status were available for all the nine cases: eight and five were positive for the estrogen and progesterone receptors (ER and PR), respectively. Seven cases had a HER2 negative tumor, and two had a positive tumor (S4 Table). These results indicated that *RECQL*-associated breast cancer was similar to those of *BRCA2*-associated breast cancer.

To test whether loss of heterozygosity (LOH) of *RECQL* occurred in the nine index cases who carried a pathogenic mutation, we performed the LOH assay in five index cases in which matched fresh tumor tissues and blood samples were available. As a result, no LOH was observed in the five cases (S5 Fig and Table 1).

## Discussion

The *RECQL* gene is located on chromosome 12p12 and encodes a protein of 649 amino-acids. It contains two important domains, the helicase domain (residues 63–418) and the RecQ carboxy-terminal (RQC) domain (residues 419–592)[7]. These domains are highly conserved in the RecQ family and are essential for helicase activity.

Five RecQ helicase proteins (named RECQL, BLM, WRN, RECQL4 and RECQL5) in humans are highly conserved and are considered to be genome caretakers that suppress neoplastic transformation[13]. Although no hereditary disease has been linked with the *RECQL* gene to date, mutations in three of five RecQ genes, *BLM*, *WRN* and *RECQ4*, lead to Bloom, Werner, and Rothmund-Thomson syndromes, respectively, and are associated with cancer predisposition and/or premature aging. One recent study suggested that germline mutations in the *BLM* gene cause susceptibility to breast cancer, although the mutations are quite rare[14]. Another study indicated that *RECQL5* polymorphisms are associated with an increased breast cancer risk in Chinese population[15].

Increasing evidence suggests that *RECQL* is involved in DNA double-strand break repair through the homologous recombination (HR) pathway[16]. RECQL-deficient cells or knockout mice exhibited chromosomal instability, sensitivity to ionizing radiation, and increased DNA damage, suggesting that *RECQL* plays an important role in the maintenance of genomic stability[17,18].

In this study, nine patients carried a pathogenic mutation in the *RECQL* gene. All of the pathogenic mutations are mapped to the above mentioned domains. The three nonsense mutations (L128X, W172X and Q266X) and one splice-site mutation (395–2 A>G) resulted in a premature truncated protein and lost the helicase activity; the five missense mutations (A195S, R215Q, R455C, M458K and T562I) were also confirmed to disrupt the function of helicase activity through a functional analysis. Therefore, mutations in the *RECQL* gene can lead to breast cancer tumorigenesis. In addition, no LOH was found in the *RECQL* mutation carriers, suggesting that *RECQL-*associated tumorigenesis may be through RECQL haploinsufficiency.

Here, we are the first to report that *RECQL* is a potential breast cancer susceptibility gene and that germline mutations in the *RECQL* gene are associated with predisposition to breast cancer. The 2.0% pathogenic mutation rate of the *RECQL* gene in familial breast cancer patients is remarkable and may be suitable for screening the mutations in *BRCA1/2-* negative breast cancer patients.

## Materials and Methods

### Study subjects

A total of 514 familial breast cancer patients were treated at the Breast Center, Peking University Cancer Hospital from 2003 to 2011. Among these, 448 index cases were negative for *BRCA1/2* germline mutations. To maximize the chance of identifying a novel breast cancer susceptibility gene, 9 unrelated early-onset familial breast cancer patients were selected from the pool of 448 cases and were subjected to whole-exome sequencing (S1 Table). These nine cases were diagnosed with breast cancer at or before the age of 35 and had at least one first-degree relative affected with breast cancer. The remaining 439 unrelated familial breast cancer patients were used to screen the potential susceptibility gene. A total of 1,588 unrelated healthy women served as controls. Approximately 95% of familial breast cancer cases and controls are ethnic Chinese Han and reside in the northern region of China. The healthy controls were age-matched to cases. Genomic DNA was extracted from peripheral blood using standard protocols for all study subjects. This study was reviewed and approved by the Ethics Committee of Peking University Cancer Hospital (project No. 2011KT12). Informed written consent was obtained from all participants.

### Whole-exome sequencing

Three micrograms of genomic DNA extracted from each blood sample was enriched for exonic regions using the SureSelect Biotinylated RNA Library (BAITS). The sequences of captured libraries were generated as 90-bp pair-end reads on an Illumina Hiseq2000. Exome capture and sequencing resulted in a minimum coverage of 10× for at least 91.3% of the capture target regions, and whole exomes were sequenced to an average mapped coverage of 108×.

### Bioinformatics analysis and variant filtration

Sequencing reads were mapped to the reference GRCh37/hg19 human genome assembly using the *Burrows-Wheeler Aligner*(*BWA*)[19]. Further processing, including duplicate removal, local realignment and base quality recalibration, was performed using Picard and *GATK*. Single nucleotide variants (SNVs) and indels were detected by *SOAPsnp*[20]and *SAMtools*, respectively. Then, filters were applied to obtain variant results of higher confidence. We then used *ANNOVAR*[21]to perform annotation and classification. The variant collection was excluded from positions found in the dbSNP 132 and 1000 Genomes databases. Only genes that harbored heterozygous variants that were truncating mutations or splice-site variants were selected for further analyses. Candidate disease-causing variants were filtered for variants affecting the same gene in at least two samples using a straightforward criterion (S2 Table). SNV and indel data were analyzed separately.

### *RECQL* mutation screening

After the filtering process, the variants in the *RECQL* gene and other potential genes found in the whole-exome sequencing assay were tested by Sanger sequencing using standard methods (S3 Table). We designed a set of 14 pairs of primers (S5 Table) to screen the entire coding regions of the *RECQL* gene in an additional 439 unrelated familial breast cancer patients and 1,588 healthy controls. The purified products were then sequenced on an ABI 3730 automated sequencer (Applied Biosystems). All mutations were confirmed in duplicate.

### RT-PCR

Total RNA was isolated from the blood samples carrying the *RECQL* c.395-2A>G mutation. Then, 2.3 μg of total RNA was transcribed to cDNA by the Superscript II Reverse Transcriptase (Invitrogen) using a random primer. PCR was then carried out using the primer pair spanning exons 3–7 of *RECQL* transcript 001 (ENST00000444129). The PCR products were separated on a 2% agarose gel by electrophoresis. The DNA fragments were re-amplified with 30 cycles, visualized and directly sequenced after gel extraction. cDNA from the blood samples of a healthy volunteer were used as the control. The analysis of c.868-12_868-11del mutation was carried out using the primer pair spanning exons 6–11 (S5 Table).

### Protein alignment and structural modeling

Multiple-sequence alignments were generated for homologous RECQL protein sequences using Clustal Omega. Jalview was used to visualize and format the alignment. Mutations in human RECQL were visualized using PyMOL on the crystal structure of the human RECQL protein (RCSB PDB, 2V1X) and the structure of this helicase in complex with a DNA substrate (RCSB PDB, 2WWY).

### Cloning, protein purification, and helicase assay

cDNA encoding full-length RECQL was cloned into the pGEX-4T-1 plasmid to be expressed as a GST fusion protein. Eight missense mutations (S63P, A195S, R215Q, R455C, M458K, H461R, R539P and T562I) were introduced by site-directed mutagenesis and confirmed by sequencing. A helicase-defective mutant K119A was produced as well and served as a negative control. Recombinant proteins were purified from BL21(DE3)-RIL cells (Stratagene) using glutathione Sepharose columns (GE Healthcare) in a buffer containing 50mM Tris-HCl (pH 7.0), 250mM NaCl, 5mM β-mercaptoethanol and concentrated to 0.2 mg/ml in another buffer containing 50mM Tris-HCl (pH 7.0), 250mM NaCl, 5mMβ-mercaptoethanol and 20% glycerol. The proteins were assayed for helicase activity, detected by the displacement of a ^32^P-5’labeled 45mer oligonucleotide from a 44mer/45mer partial DNA duplex as previously described [12]. The unwinding of the substrate DNA was detected by autoradiography and quantified by Quantity One (Bio-Rad Laboratories, Inc.). The helicase data represent the mean of three independent experiments with the mean ± S.D. indicated by error bars.

### Loss-of-heterozygosity (LOH)

Of the nine cases who carried a deleterious mutation in the *RECQL* gene, the fresh tumor tissues and blood samples were available for five cases, LOH analysis of the *RECQL* locus was carried out for these five cases. We specifically amplified the tumor DNA fragments using the same PCR conditions that we applied for germline DNA. Then, we directly sequenced them with an ABI 3730 automated sequencer (Applied Biosystems) and compared them to sequencing results of heterozygous germline DNA (S5 Fig).

### Accession codes

The following GenBank reference sequences were used for variant annotation: *RECQL*, NM_002907, *TTLL2*, NM_031949 and*VSIG2*, NM_014312.

### Web resources

1000 Genomes Project,http://browser.1000genomes.org/; dbSNP database, http://www.ncbi.nlm.nih.gov/projects/SNP/; Ensembl, http://www.ensembl.org/index.html; Picard, http://picard.sourceforge.net; GATK,http://www.broadinstitute.org/gatk/;SAMtools, http://samtools.sourceforge.net/;Clustal Omega, http://www.ebi.ac.uk/Tools/msa/clustalo/; Jalview, http://www.jalview.org/; PyMOL, http://www.pymol.org/; The Protein Data Bank, http://www.rcsb.org/pdb/.

## Supporting Information

S1 Fig Sequencing of the fifteen *RECQL* germline variants identified in 448 familial breast cancer patients. The variants are exhibited as following: (A) L128X; (B) R539P; (C) S63P; (D) c.395-2A>G; (E) W172X; (F) A195S; (G) R215Q; (H) Q266X; (I) c.868-12_868-11del; (J) R455C; (K) M458K; (L) H461R; (M) T562I; (N) M1T; (O) N363S. Recurrent variants (M1T and N363S) only show one case as an example. Variants are indicated by arrows.
(PDF)

S2 Fig Analysis of the potential splicing mutation 868–12_868-11del. RT-PCR analysis of RNA extracted from the blood sample of the affected case, using primers located in exon 6 and exon 11. The PCR products showed a normal transcript (ENST00000444129) in both the control and the patient, as confirmed by Sanger sequencing. These data showed that the 868–12_868-11del did not affect the splicing.
(PDF)

S3 Fig Stereograms of structurally important mutations in the human RECQL protein (RCSB PDB, 2V1X and 2WWY). The domains and structurally important regions in RECQL are indicated by color: the helicase domain, red; the RQC domain, blue; the β-hairpin, magenta; the ADP-binding sites, skyblue; and the conserved residues in the zinc binding subdomain, purple. The residues affected by the five missense mutations (yellow spheres) are likely to affect protein folding or helicase activity. Other objects are shown as follows: ADP, pink sticks; Mg2+, cyan sphere; Zn2+, lightblue sphere.
(PDF)

S4 Fig The pedigrees of an additional eight families with *RECQL* mutations. (A) the L128X family; (B) the W172X family; (C) the A195S family; (D) the R215Q family; (E) the Q266X family; (F) the R455C family; (G) the M458K family; (H) the H562I family. Types of cancer and age in years (y) at first diagnosis are given underneath each symbol. Filled circles represent breast cancer (BC) and double breast cancer (DBC); centre circles represent other types of cancer in the pedigrees (CerC, cervical cancer; ColC, colon cancer; EsoC, esophagus cancer; KidC, kidney cancer; LivC, liver cancer; ProC, prostate cancer). Carriers of *RECQL* mutations are shown with their specific mutations.
(PDF)

S5 Fig LOH analysis in tumor samples of five individuals carrying *RECQL* mutations. The mutations are indicated as following: (A) c.395-2A>G; (B) c.583G>T; (C) c.796C>T; (D) c.1373T>A; (E) c.1685C>T. The sequencing results from the patients’ germline and corresponding tumor DNA with the five *RECQL* mutations are shown in the upper and lower rows. All of the tumor DNAs retain heterozygosity at the *RECQL* loci.
(PDF)

S1 Table Clinical features of nine early-onset familial breast cancer patients selected for exome sequencing. (DOCX)

S2 Table Processes of single nucleotide variant filtering. (DOCX)

S3 Table List of variants that passed the filtering process. (DOCX)

S4 Table The clinical information of nine pathogenic *RECQL* mutation carriers. (DOCX)

S5 Table *RECQL* mutation analysis primers. (DOCX)
