# Supplementary material for: Mutations in RECQL Gene Are Associated with Predisposition to Breast Cancer
Source: PLoS Genet. 2015 May 6;11(5):e1005228. doi: 10.1371/journal.pgen.1005228 (PMC4422667; doi:10.1371/journal.pgen.1005228)
Supplement: S1 Fig — The variants are exhibited as following: (A) L128X; (B) R539P; (C) S63P; (D) c.395-2A>G; (E) W172X; (F) A195S; (G) R215Q; (H) Q266X; (I) c.868-12_868-11del; (J) R455C; (K) M458K; (L) H461R; (M) T562I; (N) M1T; (O) N363S. Recurrent variants (M1T and N363S) only show one case as an example. Variants are indicated by arrows. (PDF) [file pgen.1005228.s001.pdf]

S1 Fig.

A

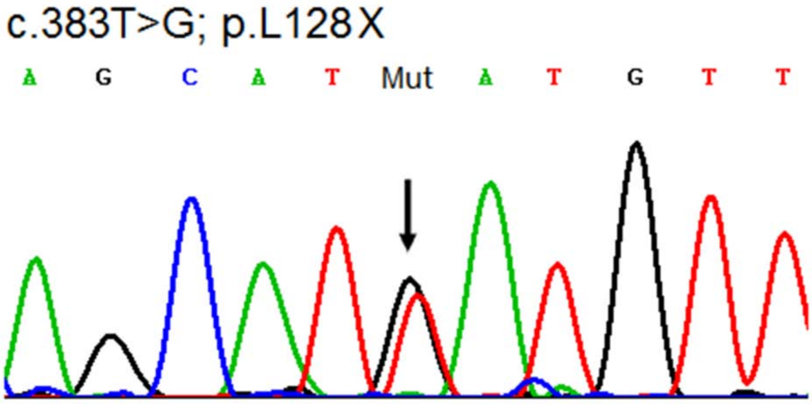
c.383T>G; p.L128X
A G C A T Mut A T G T T

B

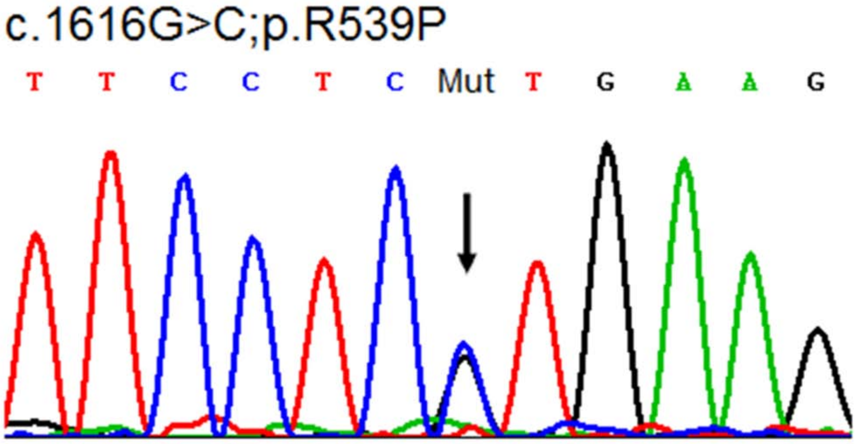
c.1616G>C;p.R539P
T T C C T C Mut T G A A G

C

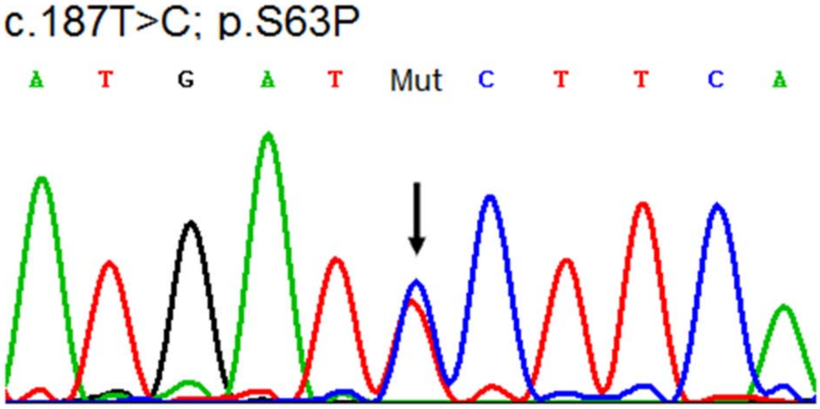
c.187T>C; p.S63P
A T G A T Mut C T T C A

D

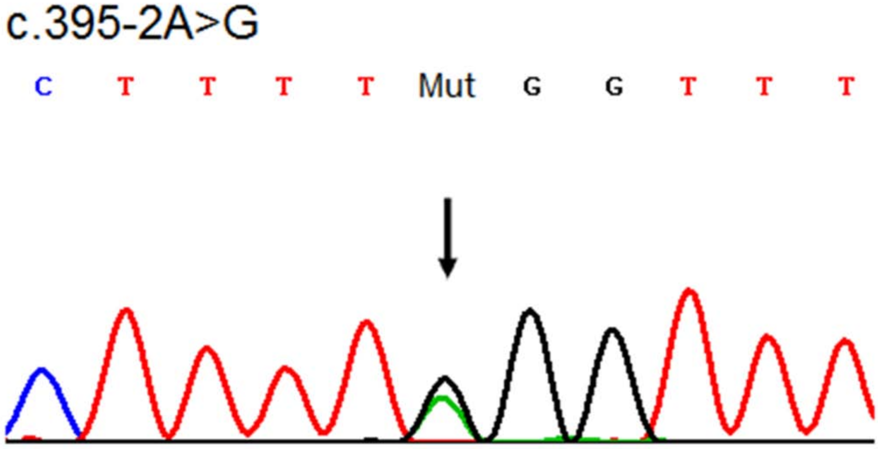
c.395-2A>G
C T T T T Mut G G T T T

E

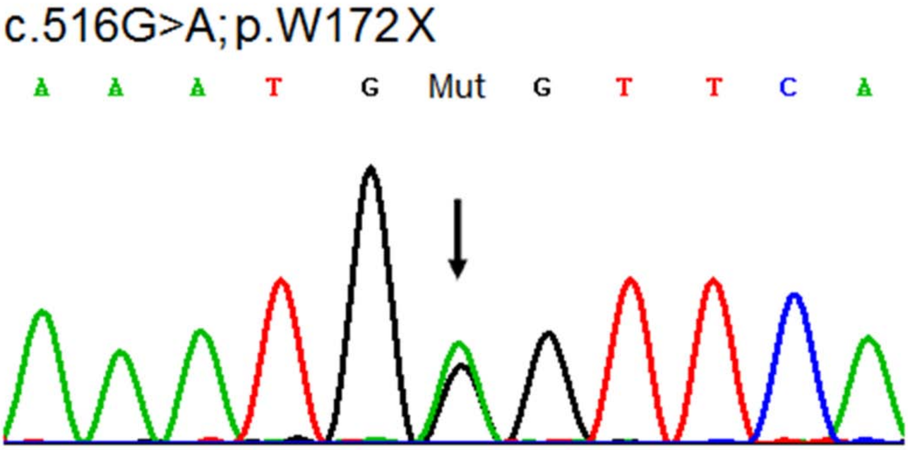
c.516G>A; p.W172 X
A A A T G Mut G T T C A

F

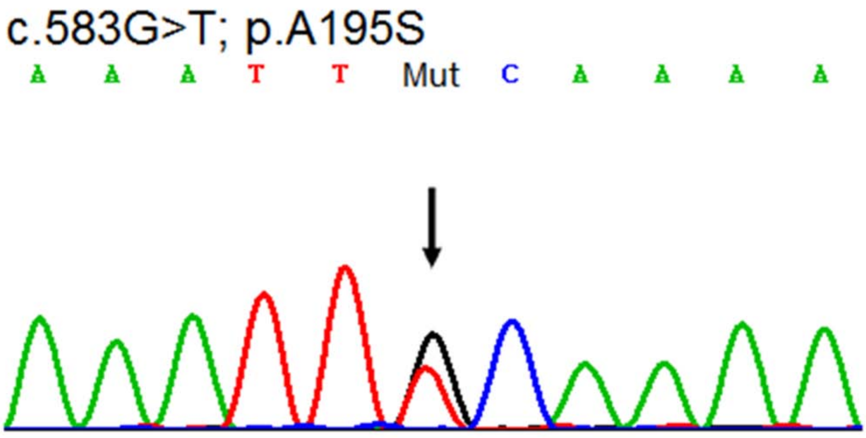
c.583G>T; p.A195S
A A A T T Mut C A A A A

G

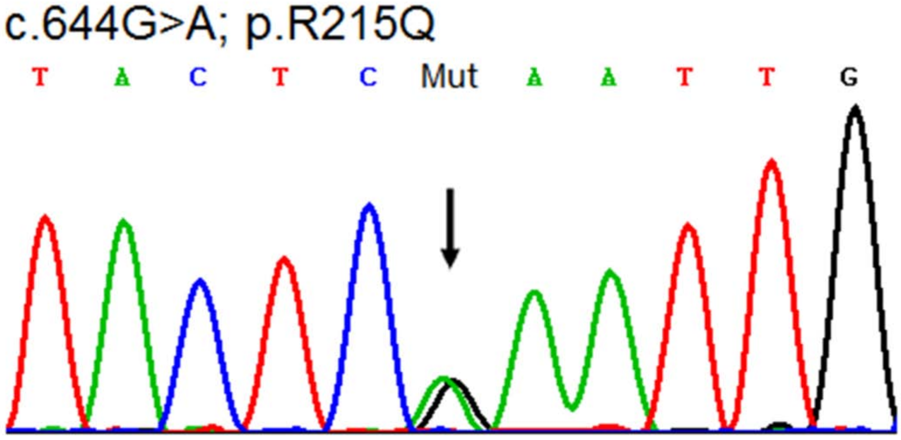
c.644G>A; p.R215Q
T A C T C Mut A A T T G

H

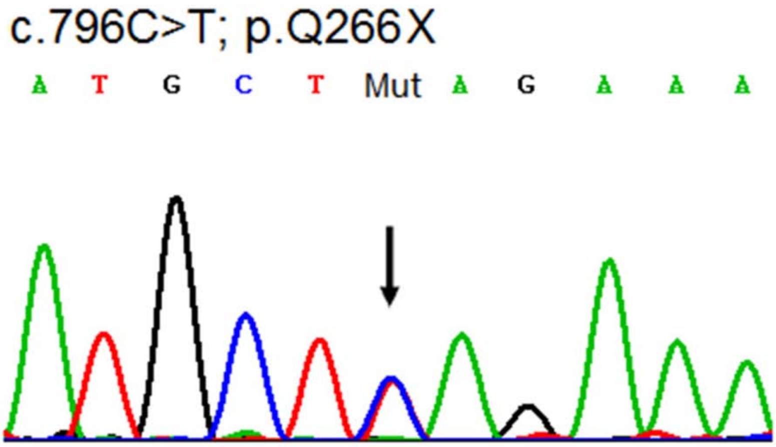
c.796C>T; p.Q266X
A T G C T Mut A G A A A

I

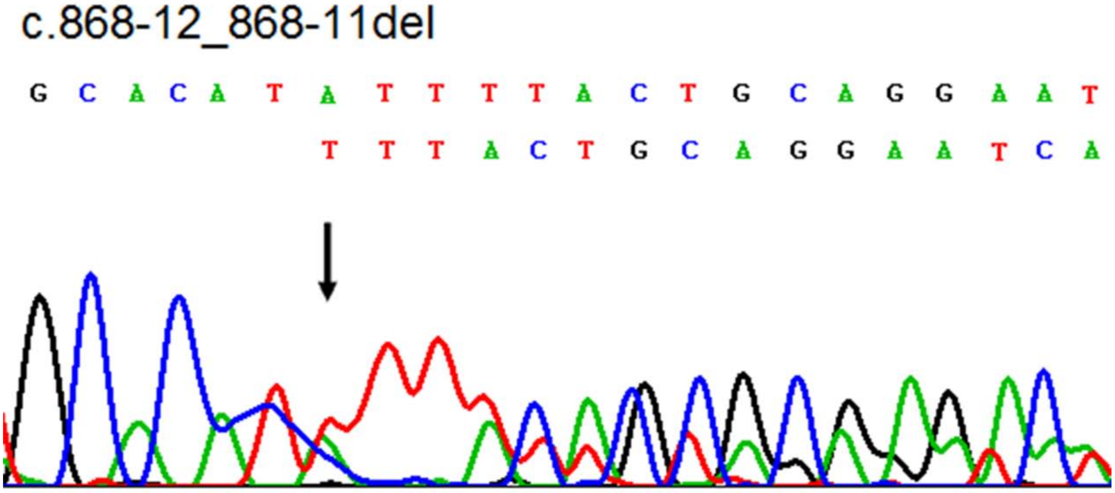
c.868-12_868-11del
G C A C A T A T T T T A C T G C A G G A A T
T T T A C T G C A G G A A T C A

J

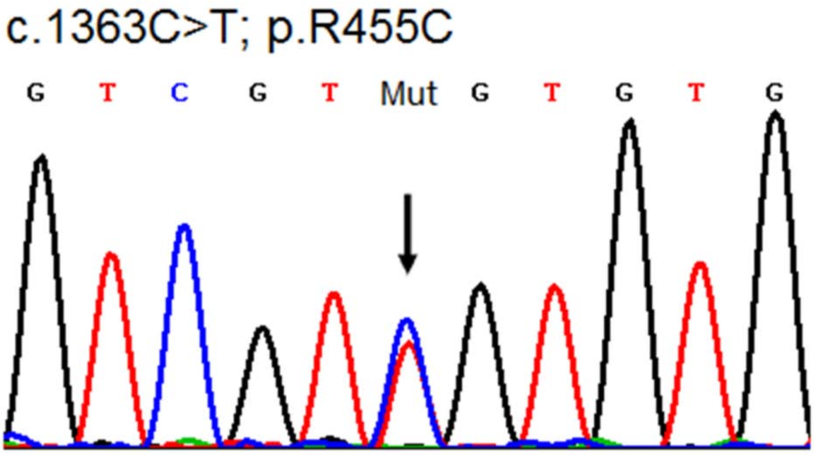

c.1363C>T; p.R455C
G T C G T Mut G T G T G

K

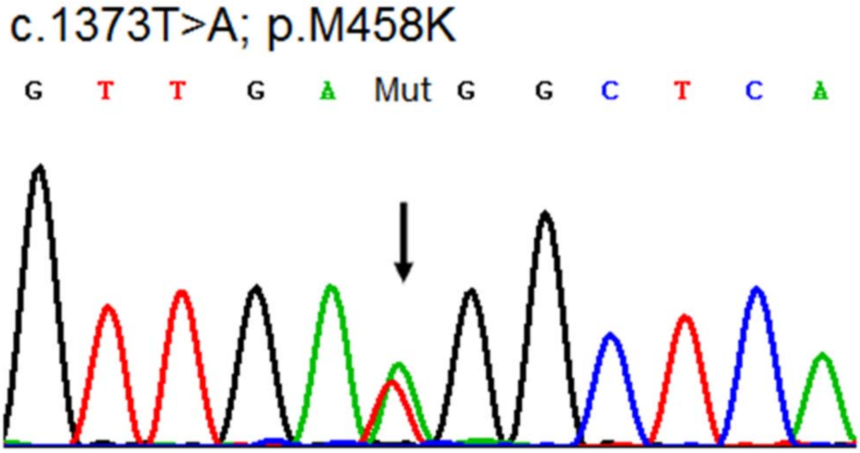

c.1373T>A; p.M458K
G T T G A Mut G G C T C A

L

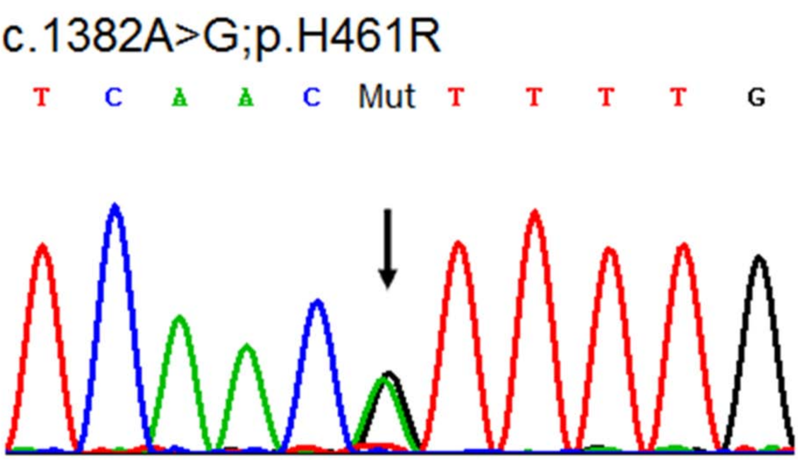

c.1382A>G;p.H461R
T C A A C Mut T T T T G

M

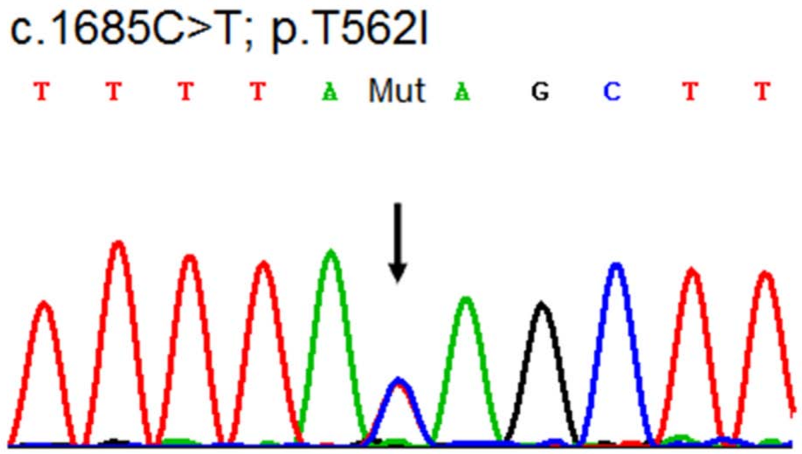
c.1685C>T; p.T562I
T T T T A Mut A G C T T

N

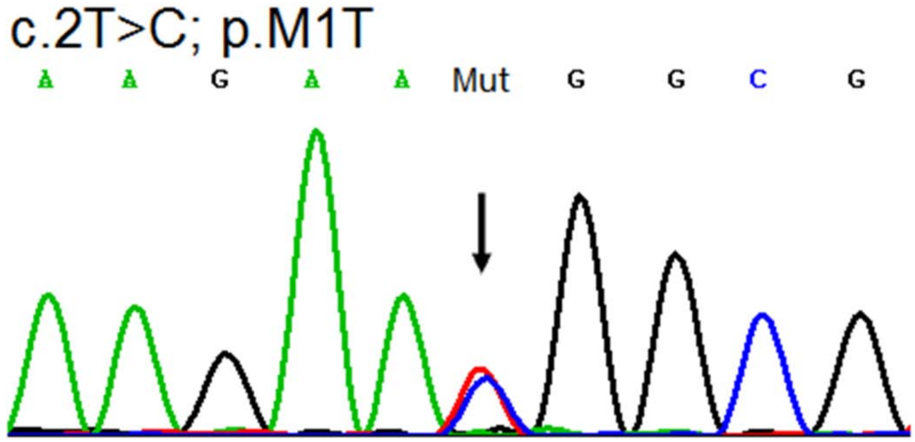
c.2T>C; p.M1T
A A G A A Mut G G C G

O

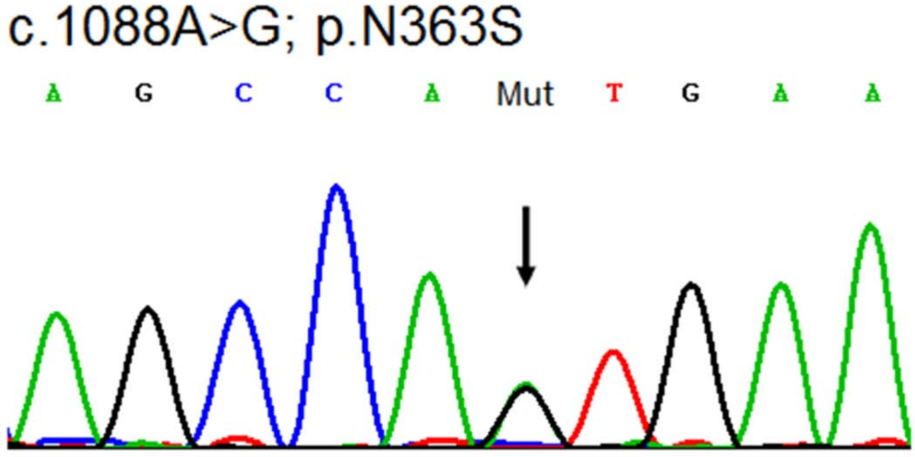
c.1088A>G; p.N363S
A G C C A Mut T G A A
