# Supplementary figures and images for: Mutations in RECQL Gene Are Associated with Predisposition to Breast Cancer
Source: PLoS Genet. 2015 May 6;11(5):e1005228. doi: 10.1371/journal.pgen.1005228 (PMC4422667; doi:10.1371/journal.pgen.1005228)

S2 Fig.

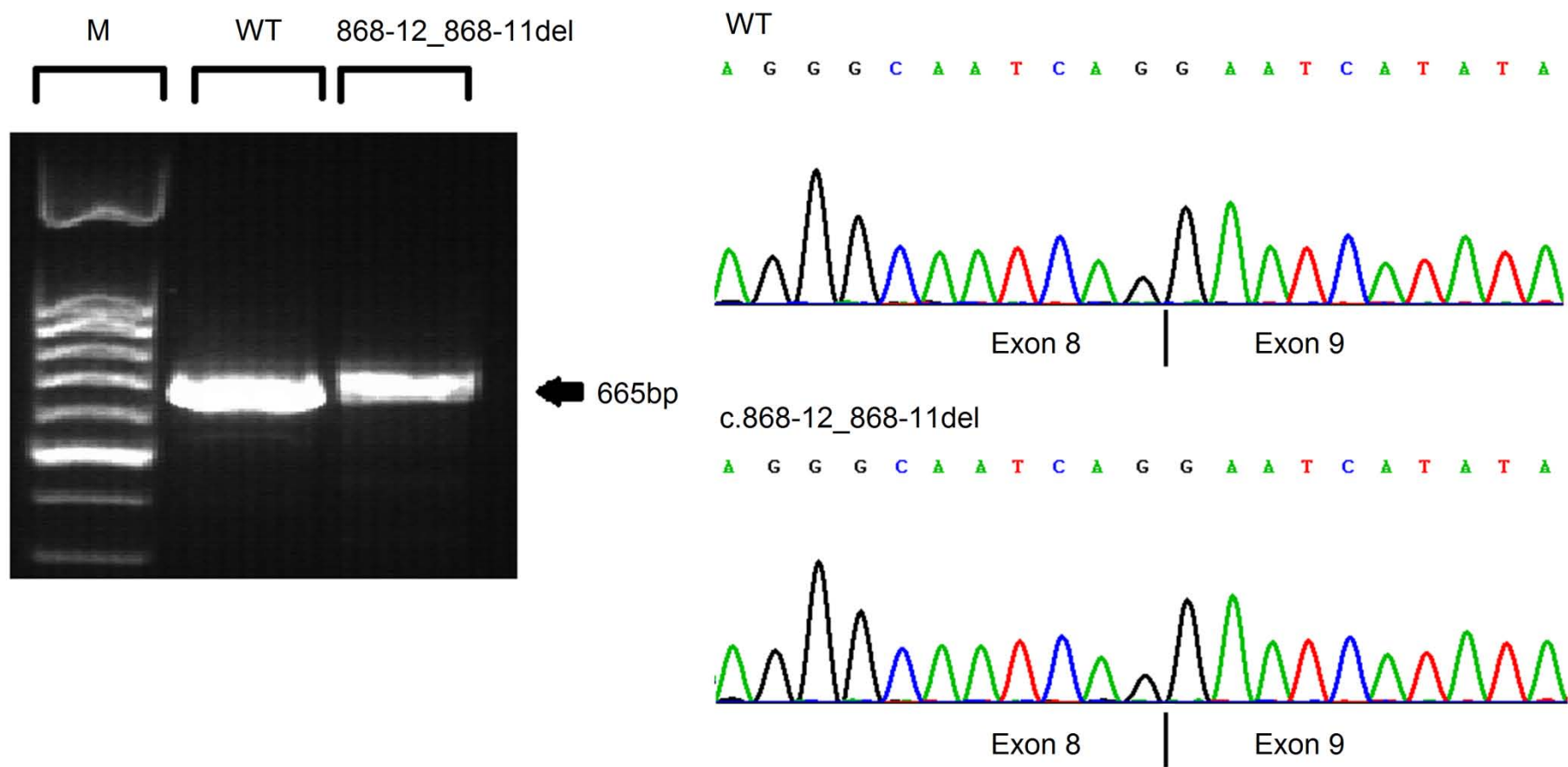

Supplement: S2 Fig — RT-PCR analysis of RNA extracted from the blood sample of the affected case, using primers located in exon 6 and exon 11. The PCR products showed a normal transcript (ENST00000444129) in both the control and the patient, as confirmed by Sanger sequencing. These data showed that the 868–12_868-11del did not affect the splicing. (PDF) [file pgen.1005228.s002.pdf]

S3 Fig.

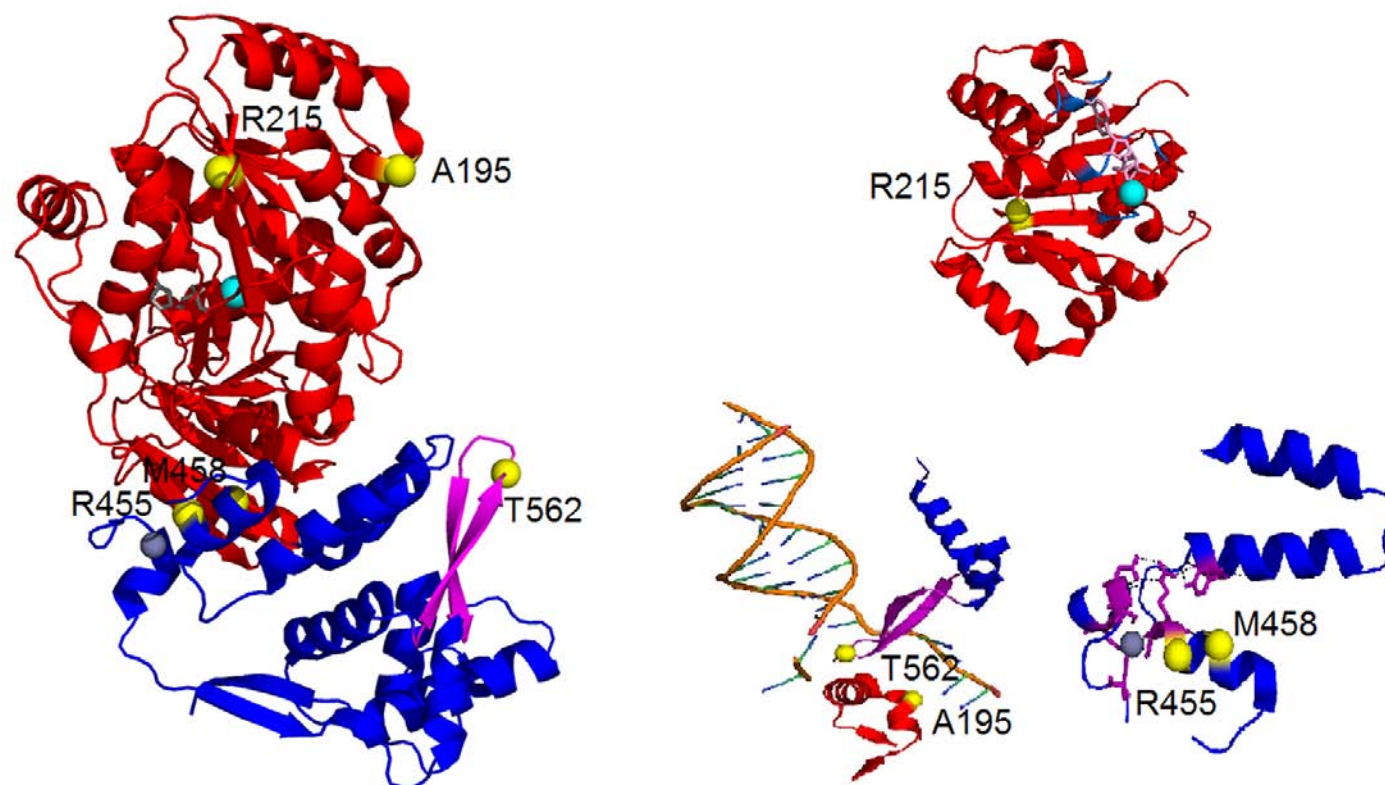

Supplement: S3 Fig — The domains and structurally important regions in RECQL are indicated by color: the helicase domain, red; the RQC domain, blue; the β-hairpin, magenta; the ADP-binding sites, skyblue; and the conserved residues in the zinc binding subdomain, purple. The residues affected by the five missense mutations (yellow spheres) are likely to affect protein folding or helicase activity. Other objects are shown as follows: ADP, pink sticks; Mg2+, cyan sphere; Zn2+, lightblue sphere. (PDF) [file pgen.1005228.s003.pdf]

S4 Fig.

A

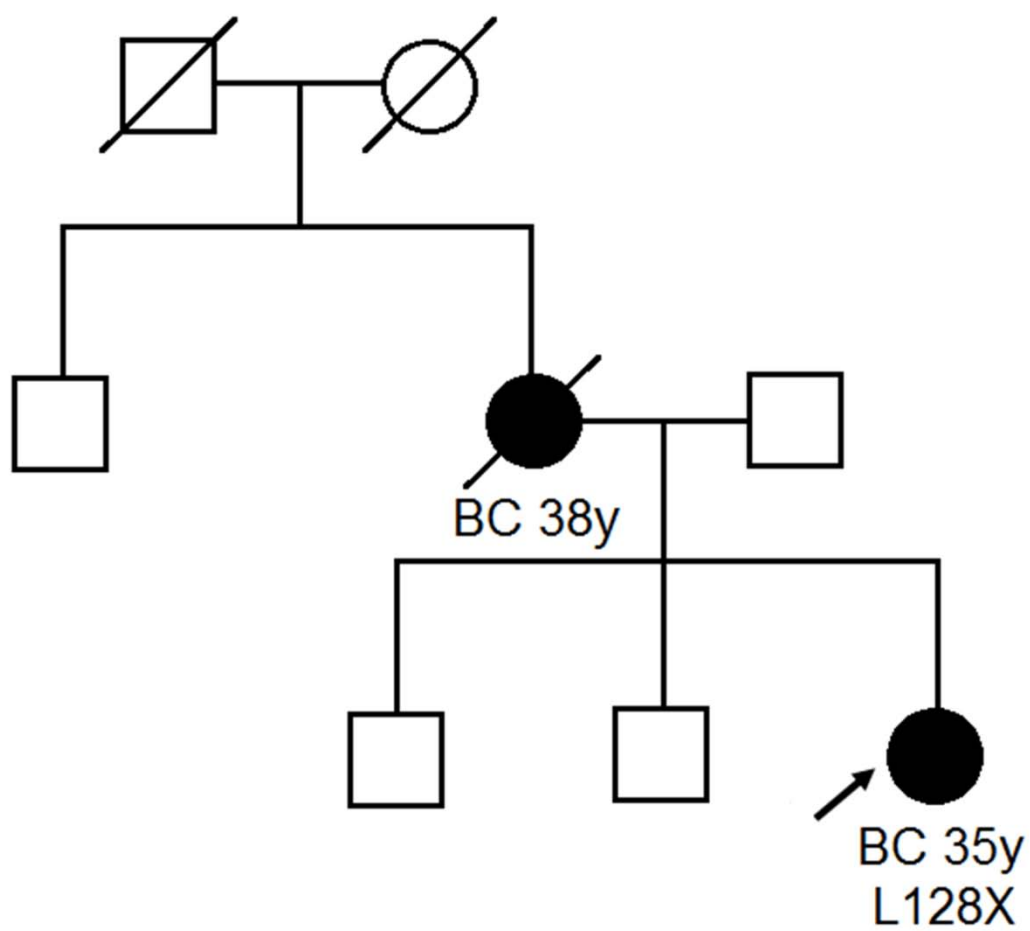

B

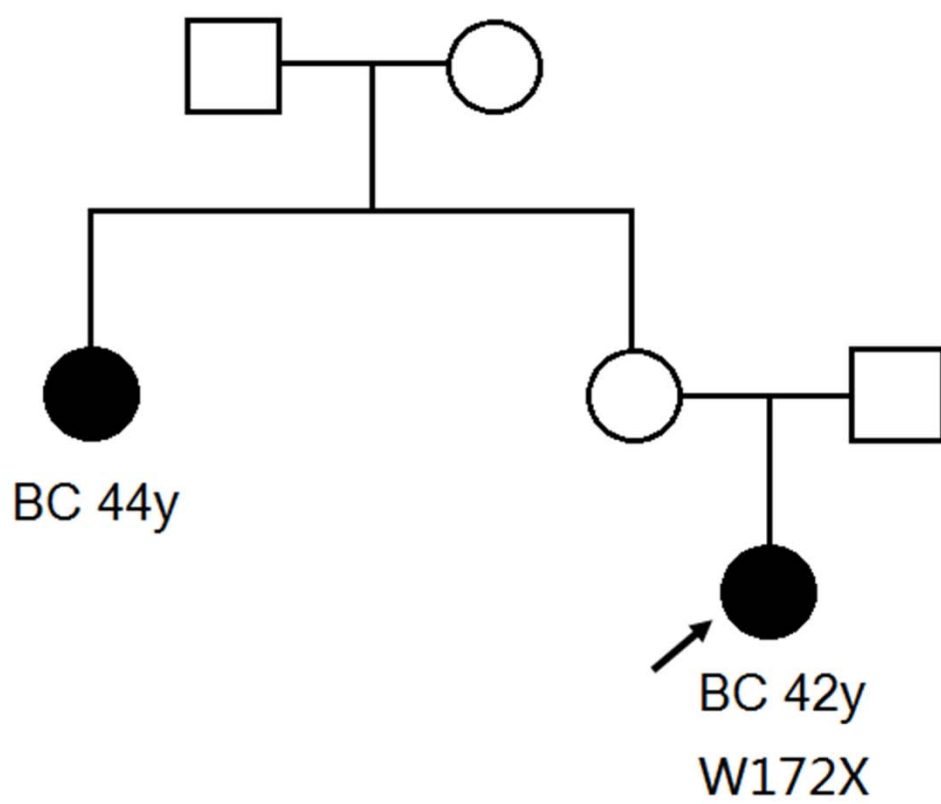

c

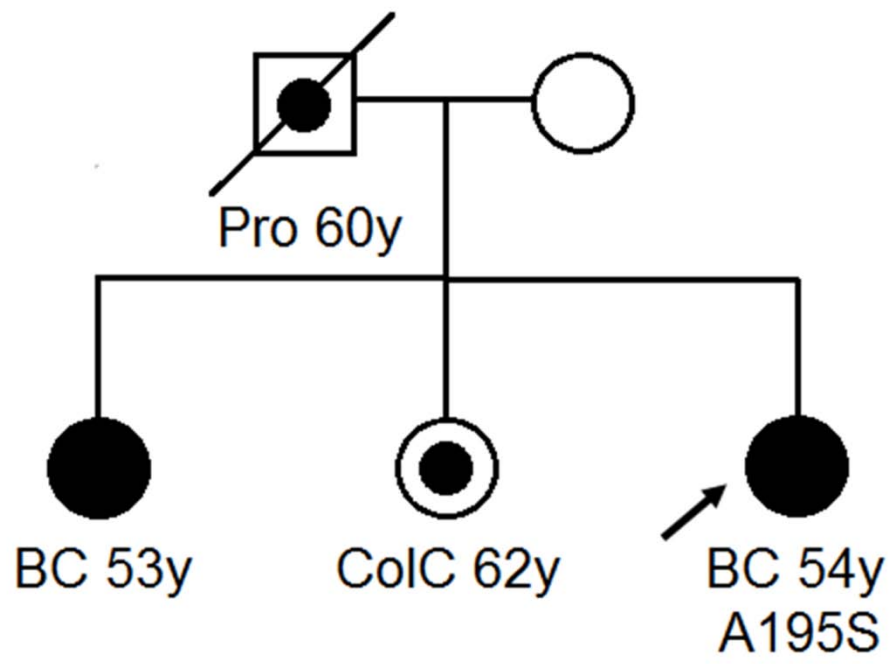

D

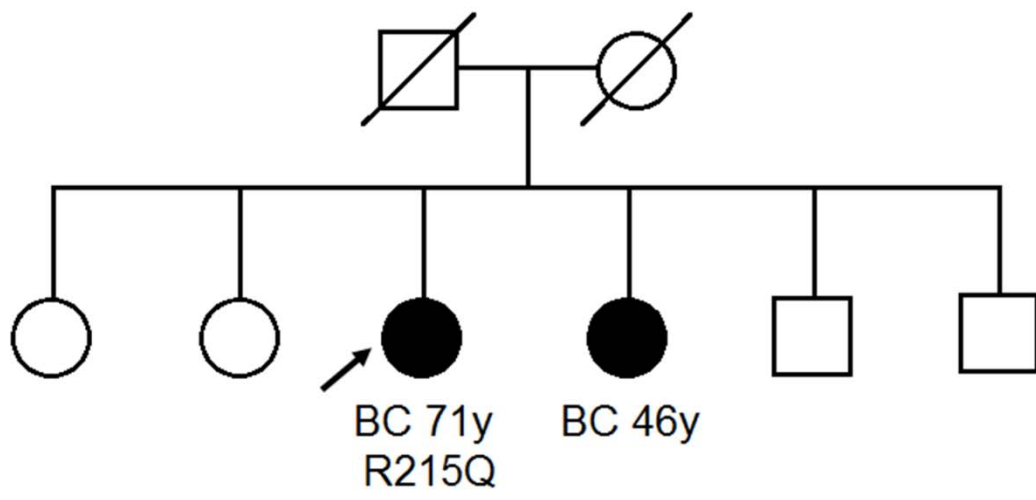

E

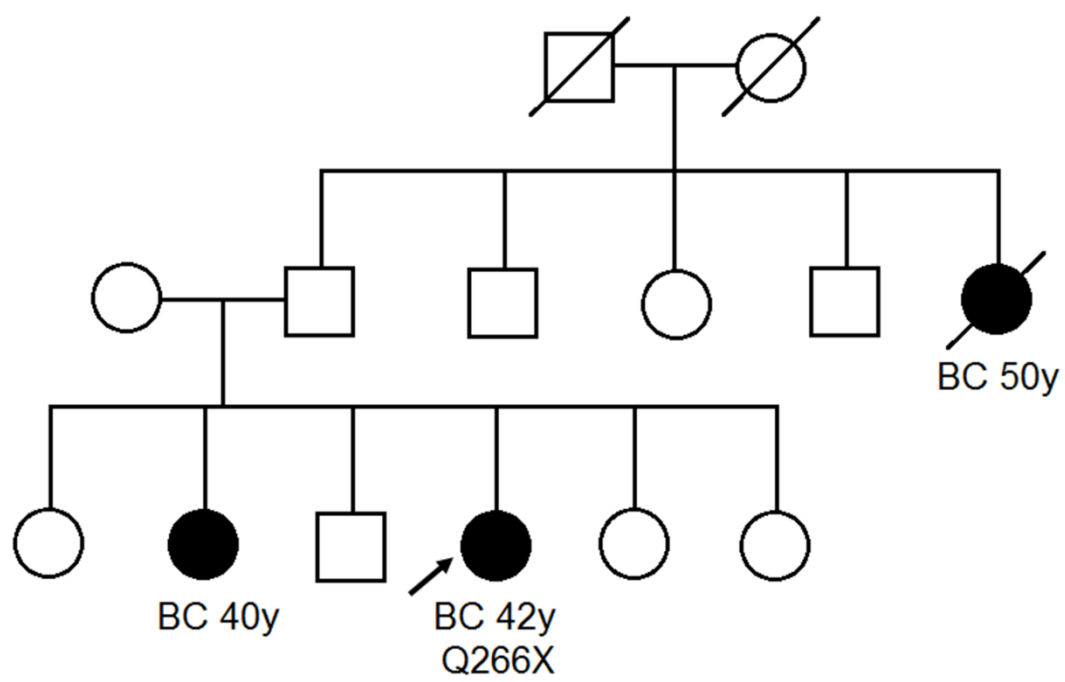

F

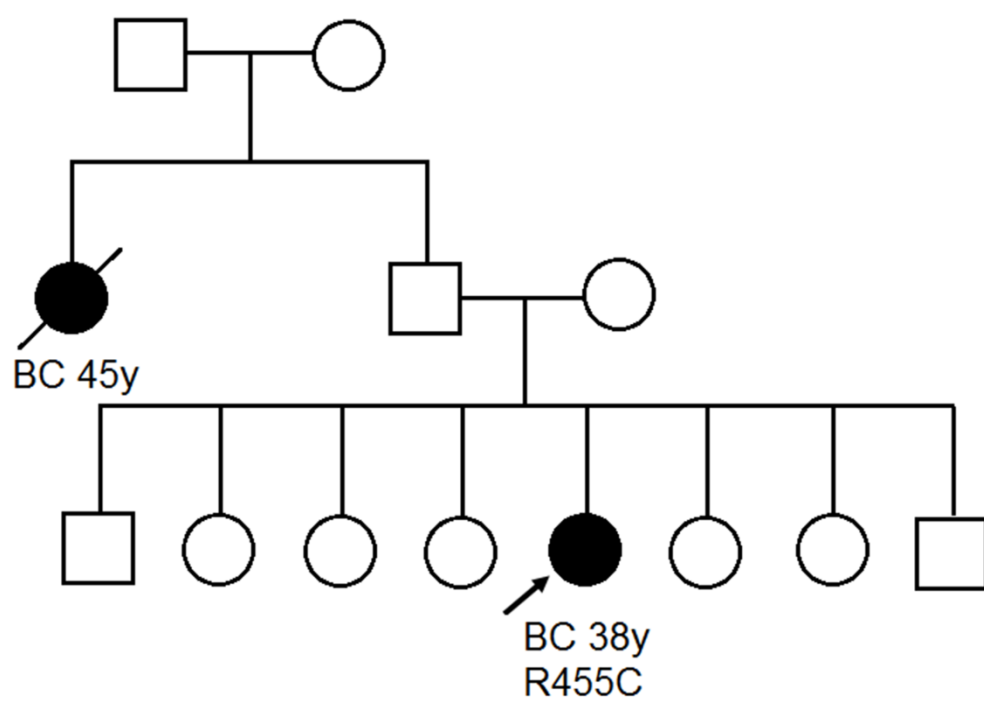

G

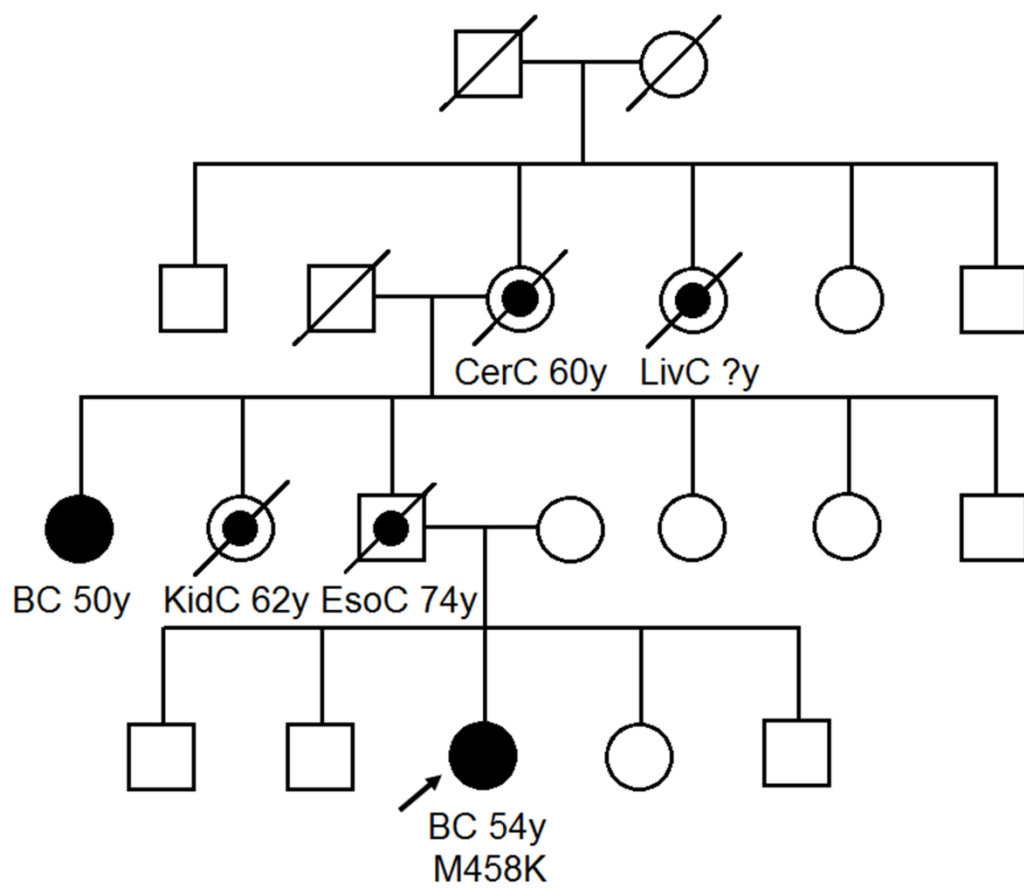

H

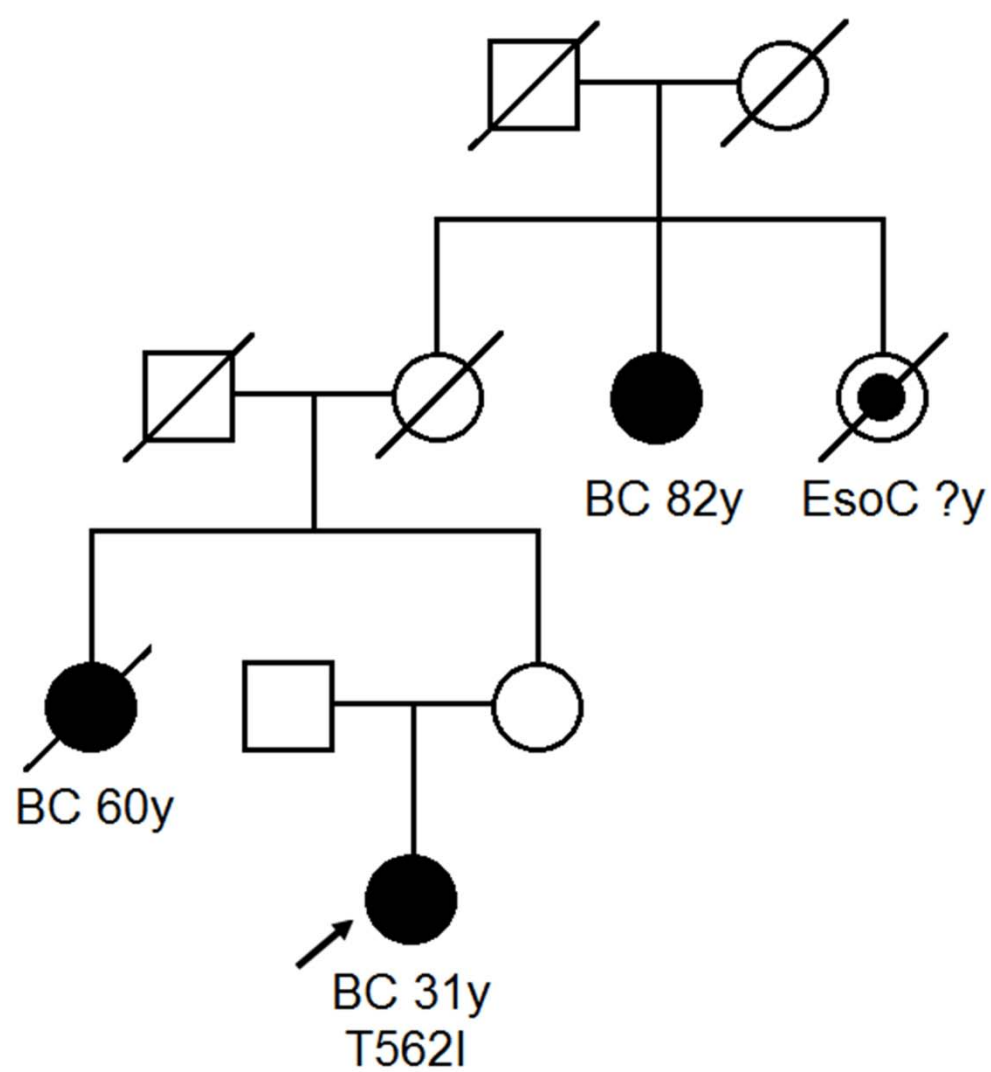

Supplement: S4 Fig — (A) the L128X family; (B) the W172X family; (C) the A195S family; (D) the R215Q family; (E) the Q266X family; (F) the R455C family; (G) the M458K family; (H) the H562I family. Types of cancer and age in years (y) at first diagnosis are given underneath each symbol. Filled circles represent breast cancer (BC) and double breast cancer (DBC); centre circles represent other types of cancer in the pedigrees (CerC, cervical cancer; ColC, colon cancer; EsoC, esophagus cancer; KidC, kidney cancer; LivC, liver cancer; ProC, prostate cancer). Carriers of RECQL mutations are shown with their specific mutations. (PDF) [file pgen.1005228.s004.pdf]
