# Supplementary material for: Mutations in RECQL Gene Are Associated with Predisposition to Breast Cancer
Source: PLoS Genet. 2015 May 6;11(5):e1005228. doi: 10.1371/journal.pgen.1005228 (PMC4422667; doi:10.1371/journal.pgen.1005228)
Supplement: S5 Fig — The mutations are indicated as following: (A) c.395-2A>G; (B) c.583G>T; (C) c.796C>T; (D) c.1373T>A; (E) c.1685C>T. The sequencing results from the patients’ germline and corresponding tumor DNA with the five RECQL mutations are shown in the upper and lower rows. All of the tumor DNAs retain heterozygosity at the RECQL loci. (PDF) [file pgen.1005228.s005.pdf]

S5 Fig.

A

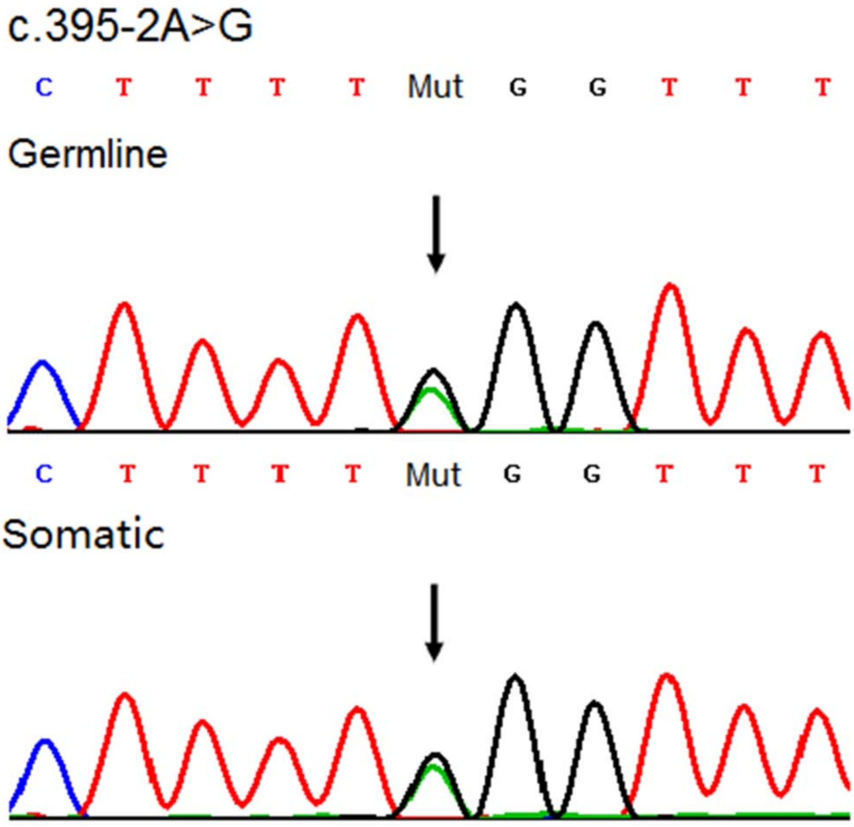
c.395-2A>G
C T T T T Mut G G T T T
Germline
C T T T T Mut G G T T T
Somatic

B

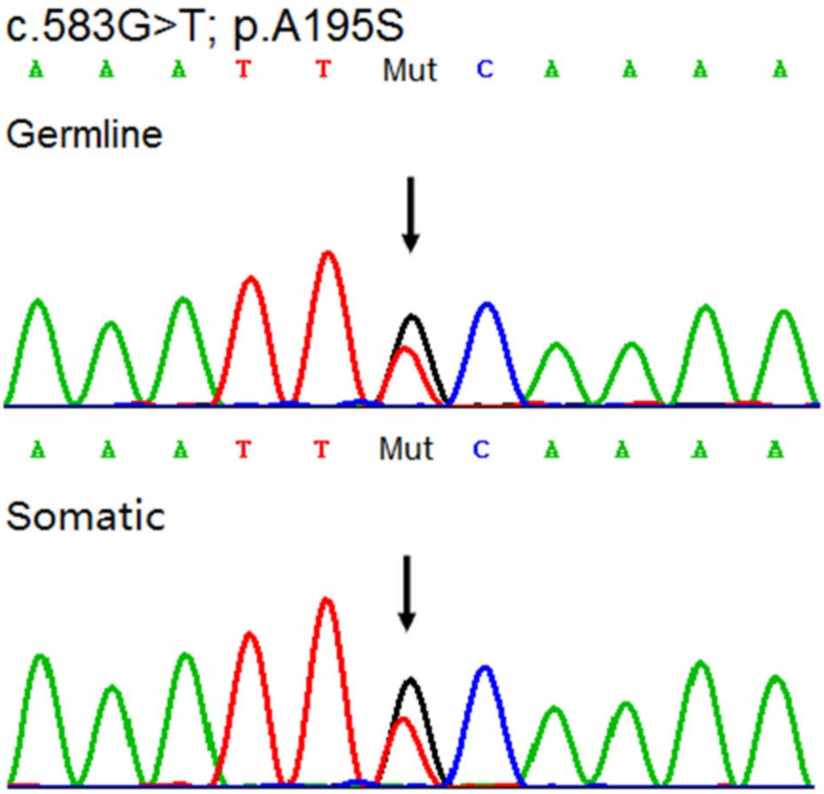
c.583G>T; p.A195S
A A A T T Mut C A A A A
Germline
A A A T T Mut C A A A A
Somatic

C

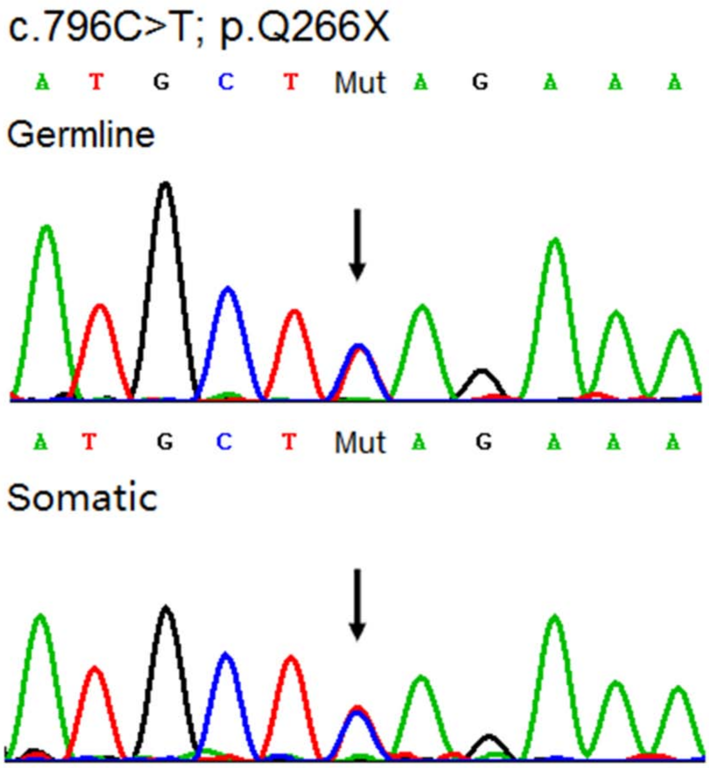

c.796C>T; p.Q266X
A T G C T Mut A G A A A
Germline
A T G C T Mut A G A A A
Somatic

D

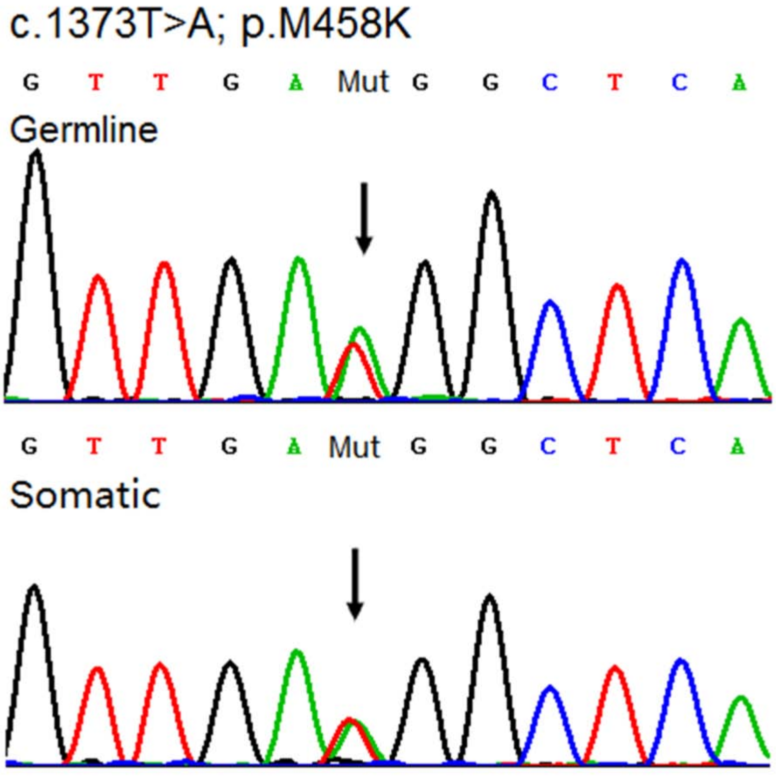

c.1373T>A; p.M458K
G T T G A Mut G G C T C A
Germline
G T T G A Mut G G C T C A
Somatic

E

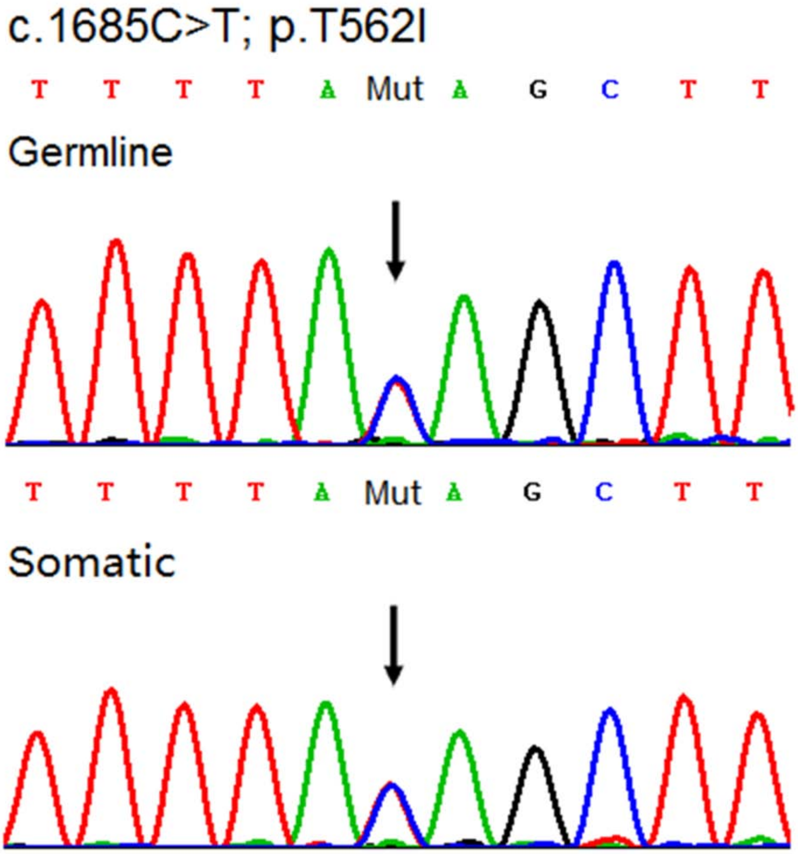
c.1685C>T; p.T562I
T T T T A Mut A G C T T
Germline
T T T T A Mut A G C T T
Somatic
