# Supplementary material for: Mutations in RECQL Gene Are Associated with Predisposition to Breast Cancer
Source: PLoS Genet. 2015 May 6;11(5):e1005228. doi: 10.1371/journal.pgen.1005228 (PMC4422667; doi:10.1371/journal.pgen.1005228)
Supplement: S1 Table — (DOCX) [file pgen.1005228.s006.docx]

| **S1 Table.** Clinical features of nineearly-onsetfamilial breast cancer patients selected for exome sequencing | | | | |
| --- | --- | --- | --- | --- |
| Family | Patient ID | Age at onset^a^ | Breast cancer affected family member and age | Other cancer types in the family |
| 1 | 7004 | DBC, 22&29 | mother, 35; grandmother, unknown | — |
| 2 | 5148 | DBC, 35&38 | mother, 32 | — |
| 3 | 2597 | BC, 31 | mother, 52 | stomach |
| 4 | 3299 | BC, 31 | mother, 52 | — |
| 5 | 5034 | BC, 25 | mother, 46 | — |
| 6 | 6077 | BC, 29 | mother, 54 | — |
| 7 | 6514 | BC, 35 | mother, 38 | — |
| 8 | 6599 | BC, 35 | sister, 42; maternal aunt, 65 | — |
| 9 | 6810 | BC, 25 | mother, 45 | colorectal |
| ^a^Double breast cancer (DBC), breast cancer (BC). | | | | |
