# Supplementary material for: Mutations in RECQL Gene Are Associated with Predisposition to Breast Cancer
Source: PLoS Genet. 2015 May 6;11(5):e1005228. doi: 10.1371/journal.pgen.1005228 (PMC4422667; doi:10.1371/journal.pgen.1005228)
Supplement: S2 Table — (DOCX) [file pgen.1005228.s007.docx]

| **S2 Table.** Processes of single nucleotide variant filtering | | | | | | | | | |
| --- | --- | --- | --- | --- | --- | --- | --- | --- | --- |
| Sample ID | Total number of SNVs | SNVs after 1000 genome and dbSNP 132 filtering | SNVs in coding regions and splice sites | SNVs after removing synonymous variants | Heterozygous SNVs | Different SNVs affecting the same gene in at least two samples | SNVs including one or more nonsense mutation | SNVs after the last version of 1000 genomes and dbSNP 138 filtering | SNVs after filtering mapping errors not validated |
| 7004 | 43,694 | 1,791 | 825 | 544 | 534 |  |  |  |  |
| 5148 | 44,017 | 1,872 | 866 | 545 | 540 |  |  |  |  |
| 2597 | 43,268 | 1,727 | 828 | 518 | 513 |  |  |  |  |
| 3299 | 43,401 | 1,742 | 818 | 545 | 539 |  |  |  |  |
| 5034 | 43,817 | 1,886 | 862 | 584 | 575 |  |  |  |  |
| 6077 | 43,429 | 1,732 | 743 | 495 | 492 |  |  |  |  |
| 6514 | 44,531 | 2,037 | 976 | 625 | 621 |  |  |  |  |
| 6599 | 43,210 | 1,752 | 799 | 495 | 486 |  |  |  |  |
| 6810 | 43,708 | 1,890 | 895 | 593 | 581 |  |  |  |  |
| Average | 43,675 | 1,825 | 846 | 549 | 542 | 1124 | 82 | 23 | 6 |
| Percent of SNVs remaining | 100 | 4.18 | 1.94 | 1.26 | 1.24 | 2.57 | 0.19 | 0.05 | 0.01 |
