# Supplementary material for: Mutations in RECQL Gene Are Associated with Predisposition to Breast Cancer
Source: PLoS Genet. 2015 May 6;11(5):e1005228. doi: 10.1371/journal.pgen.1005228 (PMC4422667; doi:10.1371/journal.pgen.1005228)
Supplement: S3 Table — (DOCX) [file pgen.1005228.s008.docx]

| **S3 Table.** List of variants that passed the filtering process | | | | | | | | |
| --- | --- | --- | --- | --- | --- | --- | --- | --- |
| Chr | Position | Ref | Alt | Gene | ExonicFunc | NM_RefSeq | Amino acid change | Sample ID |
| 6 | 167754871 | C | T | TTLL2 | stopgain SNV | NM_031949 | p.Q495X | 5034 |
| 6 | 167754144 | T | G | TTLL2 | stopgain SNV | NM_031949 | p.Y252X | 6599 |
| 11 | 124617531 | T | A | VSIG2 | nonsynonymous SNV | NM_014312 | p.H295L | 7004 |
| 11 | 124617493 | C | A | VSIG2 | stopgain SNV | NM_014312 | p.E308X | 6599 |
| 12 | 21624413 | C | G | RECQL | nonsynonymous SNV | NM_002907 | p.R539P | 5034 |
| 12 | 21643144 | A | C | RECQL | stopgain SNV | NM_002907 | p.L128X | 6514 |
