# Supplementary material for: Mutations in RECQL Gene Are Associated with Predisposition to Breast Cancer
Source: PLoS Genet. 2015 May 6;11(5):e1005228. doi: 10.1371/journal.pgen.1005228 (PMC4422667; doi:10.1371/journal.pgen.1005228)
Supplement: S4 Table — (DOCX) [file pgen.1005228.s009.docx]

| **S4 Table.** The clinical information of nine pathogenic *RECQL* mutation carriers | | | | | | | | | | |
| --- | --- | --- | --- | --- | --- | --- | --- | --- | --- | --- |
| ID | Coding mutation^a^ | Protein change | Age at diagnosis, y | Tumor size, cm | Histology | Grade | ER | PR | HER2 | Lymph node status |
| 6514 | 383T>G | L128X | 35 | 1.4 | IDC | 3 | - | - | - | - |
| 2368 | 516G>A | W172X | 42 | 1.4 | IDC | 2 | + | + | - | + |
| 5435 | 796C>T | Q266X | 42 | 1.9 | IDC | 2 | + | + | - | + |
| 955 | 395-2A>G | G132fs* | 41 | 2.1 | IDC | 2 | + | - | - | + |
| 2931 | 583G>T | A195S | 52 | 1.2 | IDC | 3 | + | - | + | - |
| 5570 | 644G>A | R215Q | 71 | 0.9 | IDC | 2 | + | + | - | - |
| 4212 | 1363C>T | R455C | 38 | 1.6 | IDC | 2 | + | + | - | - |
| 6089 | 1373T>A | M458K | 54 | 2.0 | IDC | 2 | + | + | - | - |
| 2943 | 1685C>T | T562I | 31 | 2.3 | IDC | 2 | + | - | + | - |
| ER: estrogen receptor; PR: progesterone receptor; IDC: invasive ductal carcinoma;  ^a^The reference transcript is *RECQL* transcript 001 (ENST00000444129); | | | | | | | | | | |
