# Supplementary material for: Mutations in RECQL Gene Are Associated with Predisposition to Breast Cancer
Source: PLoS Genet. 2015 May 6;11(5):e1005228. doi: 10.1371/journal.pgen.1005228 (PMC4422667; doi:10.1371/journal.pgen.1005228)
Supplement: S5 Table — (DOCX) [file pgen.1005228.s010.docx]

| **S5 Table.** *RECQL* mutation analysis primers | | |
| --- | --- | --- |
|  | Name | Primer sequence 5’-3’ |
| DNA | *RECQL* ex 2F | 5'- AGTCGTTCTGAGTGTTGTGG -3' |
|  | *RECQL* ex 2R | 5'- GGTTAGCATGGCAAAGTTTGTA -3' |
|  | *RECQL* ex 3F | 5'- GAGGTCACTCAGCAGGTAAAAGG -3' |
|  | *RECQL* ex 3R | 5'- CTCTAAGCCTACTGGAAAGCACTG -3' |
|  | *RECQL* ex 4F | 5'- AGCCACCATGCCTACTATGTC -3' |
|  | *RECQL* ex 4R | 5'- GATGACAAAGCACTTCTTCAACTCA -3' |
|  | *RECQL* ex 5-1F | 5'- CTGAATTGATGGCCAGTGCC -3' |
|  | *RECQL* ex 5-1R | 5'- TGCCACTGAAACATACCTTAGAAC -3' |
|  | *RECQL* ex 5-2F | 5'- TTTTTCTCTTTTAGGTTTTACACTCG -3' |
|  | *RECQL* ex 5-2R | 5'- CTGCATAATTAGCAATTCTCCCT -3' |
|  | *RECQL* ex 6F | 5'- ACAGCTGCCATGATGTTTCGT -3' |
|  | *RECQL* ex 6R | 5'- ACAGACTACAGTGGGGAATGAAAC -3' |
|  | *RECQL* ex 7F | 5'- TTGAGTCCACCGTGACCAAGAG -3' |
|  | *RECQL* ex 7R | 5'- GCAGATCCCCTCTGCGTAAT -3' |
|  | *RECQL* ex 8F | 5'- TAGACAGCCACTTGGATGGTACT -3' |
|  | *RECQL* ex 8R | 5'- AGGCATAAGCAAATGTCACACCC -3' |
|  | *RECQL* ex 9&10F | 5'- TTAATCAATGTCCTGAATGTGTGTG -3' |
|  | *RECQL* ex 9&10R | 5'- TTTCTCCATATGCAAGTAAGTGTCA -3' |
|  | *RECQL* ex 11F | 5'- AGATGTAGTATAAAACATTGCCTGC -3' |
|  | *RECQL* ex 11R | 5'- TACAGGTTGATGTGCTGGTTCC -3' |
|  | *RECQL* ex 12F | 5'- GATTGTGTGATGTGAGAAGAACCTG -3' |
|  | *RECQL* ex 12R | 5'- TGGACCACGTATATGCCAGTG -3' |
|  | *RECQL* ex 13F | 5'- GTGGCTCACATTGATAACCAGC -3' |
|  | *RECQL* ex 13R | 5'- CACACAAAATAACTGCAAAACCGT -3' |
|  | *RECQL* ex 14&15F | 5'- ATTGCAACAGAATAAACGGTTTTG -3' |
|  | *RECQL* ex 14R | 5'- TATTCAAACGGAGTCCTCCCA -3' |
|  | *RECQL* ex 15R | 5'- CTCAAAAGTTTAGATCTTCAGAGATAAG -3' |
| cDNA | *RECQL* SP1 F | 5'- TGATTCTTCACCTGCCGCTTG -3' |
|  | *RECQL* SP1 R | 5'- TTGTTGCAGTTGCAGTCAGCC -3' |
|  | *RECQL* SP2 F | 5’- GTTCACTGCTGTAGTCAGTGGGG -3’ |
|  | *RECQL* SP2 R | 5’- TCATAAAGCTTCTGCTGTCCCACA -3’ |
